# Case Report: Simultaneous continuous renal replacement therapy and endovascular coiling in a poor-grade ruptured distal anterior cerebral artery aneurysm with ESKD and life-threatening hyperkalemia

**DOI:** 10.3389/fsurg.2026.1997449

**Published:** 2026-10-23

**Authors:** Zhifu Song, Yulan Gan, Xueping Huang, Wenyou Wu

**Affiliations:** 1 Department of Cerebrovascular Disease, Luzhou People’s Hospital, Luzhou, Sichuan, China; 2 Department of Neonatology, Luzhou People’s Hospital, Luzhou, Sichuan, China; 3 Department of Neurosurgery, Luzhou People’s Hospital, Luzhou, Sichuan, China

**Keywords:** case report, continuous renal replacement therapy, end-stage kidney disease, hyperkalemia, poor-grade subarachnoid hemorrhage

## Abstract

**Background:**

Poor-grade aneurysmal subarachnoid hemorrhage (aSAH) in patients with end-stage kidney disease (ESKD) on maintenance hemodialysis has traditionally been considered futile. Uremic platelet dysfunction and coagulopathy, electrolyte disturbances, and poor multi-organ reserve make these patients poor candidates for aggressive intervention. When a ruptured aneurysm coexists with life-threatening hyperkalemia, the therapeutic window narrows further, because severe hyperkalemia significantly increases the risk of perioperative arrhythmia and cardiac arrest and requires urgent correction. Continuous renal replacement therapy (CRRT) may offer advantages in this setting, but its use during neurointerventional procedures has rarely been reported.

**Case report:**

A 43-year-old woman with polycystic kidney disease and ESKD on regular hemodialysis presented with sudden coma and was diagnosed with Hunt-Hess grade V aSAH from a ruptured distal anterior cerebral artery (DACA) aneurysm. After 5 days of intensive care, her neurological status improved sufficiently to consider intervention. However, she developed life-threatening hyperkalemia (potassium 7.6 mmol/L). We performed simultaneous continuous renal replacement therapy (CRRT) and endovascular coiling. No anticoagulation was used in the CRRT circuit; intravenous heparin 0.67 mg/kg was administered separately for the neurointerventional procedure. CRRT was delivered in CVVHDF mode with a net ultrafiltration rate of 1−2 mL/kg/h and a total clearance of approximately 57 mL/kg/h, maintaining hemodynamic and intracranial stability throughout the 2-hour procedure. The aneurysm was completely obliterated (Raymond class I), and potassium decreased to 5.2 mmol/L. The patient recovered neurologically postoperatively but developed refractory bleeding during tracheostomy due to uremic platelet dysfunction and coagulopathy, ultimately leading to airway complications and death at 3 months.

**Conclusion:**

Simultaneous CRRT and endovascular coiling was technically feasible in this carefully selected patient with poor-grade aSAH, ESKD, and life-threatening hyperkalemia. However, this is a single-case experience and cannot be generalized, and perioperative bleeding and airway management remained challenging.

## Introduction

Poor-grade aneurysmal subarachnoid hemorrhage (Hunt-Hess IV-V) carries high mortality. When ESKD coexists, these vulnerable patients are often considered to have no treatment value. Uremic platelet dysfunction and coagulopathy, together with electrolyte disturbances and poor multi-organ reserve, lead many centers to exclude these patients from active intervention (1, 2).

**Table 1:** Timeline of clinical events.

Time	Neurological Status	Major Events	Interventions	Potassium (mmol/L)
Day 1 (admission)	GCS 3, Hunt-Hess V, anisocoria, absent light reflexes	Sudden coma; family refused surgery	Intubation, mechanical ventilation, mannitol, levetiracetam, nimodipine; continuous sedation-analgesia (propofol + sufentanil + midazolam)	4.7
Day 2	Pupils became isometric	Hypotension (SBP nadir 70 mmHg)	Dopamine + isoproterenol infusion; bedside hemodialysis	-
Day 6 (morning)	GCS 7 (E2V1M4), Hunt-Hess IV, isometric pupils with sluggish light reflex	Family consented to surgery; CTA confirmed lobulated aneurysm at left A3 segment	Sedatives discontinued; neurological assessment	6.6 → 7.6
Day 6 (afternoon)	(intraoperative, no formal GCS assessment)	Preoperative hyperkalemia (7.6 mmol/L)	Simultaneous CRRT and endovascular coiling; complete aneurysm obliteration (Raymond class I)	7.6 → 5.2
Day 8	Alert, could follow commands	Refractory bleeding during tracheostomy	Platelet transfusion, tranexamic acid; procedure aborted	-
Postoperative weeks 2–3	Excellent neurological function	Airway scarring, crust formation, recurrent obstruction	Multidisciplinary airway management: tube changes, enhanced humidification, bronchoscopic clearance	-
Postoperative month 3	(terminal event)	Acute airway obstruction	Death	-

Polycystic kidney disease is a common cause of ESKD. The 2025 KDIGO guideline states that the prevalence of intracranial aneurysm in ADPKD patients is approximately 12.9%, 4- to 5-fold higher than the general population, and recommends screening for patients with a personal history of SAH or a family history of IA/SAH/unexplained sudden death who are candidates for treatment, with non-contrast MRA as the preferred modality (3). Distal anterior cerebral artery (DACA) aneurysms are technically challenging for both surgical and endovascular approaches.

When a ruptured aneurysm coexists with life-threatening hyperkalemia, decision-making becomes extremely difficult. IHD can rapidly correct potassium, but it may cause rapid osmotic shifts and has a significant impact on intracranial pressure and hemodynamics, which is particularly hazardous in a patient with cerebral herniation and coagulopathy. In addition, a standard IHD session requires 3−4 h, during which the aneurysm remains unprotected and the re-rupture risk is substantial. The POGASH registry reported a 17.6% pre-treatment rebleeding rate in poor-grade SAH (4). CRRT was chosen in this patient because of the need for continuous potassium control, hemodynamic stability, and avoidance of rapid osmotic shifts in the setting of cerebral herniation and coagulopathy. This case illustrates a possible integrated approach in which CRRT is performed during the endovascular procedure, which may offer a treatment option for selected patients who would otherwise be considered untreatable.

To our knowledge, no prior report has described simultaneous CRRT and endovascular coiling in a patient with poor-grade aSAH and ESKD. A literature search of PubMed, Embase, and Web of Science using the terms (CRRT OR continuous renal replacement therapy) AND (coiling OR endovascular coiling) AND (intracranial aneurysm OR subarachnoid hemorrhage) AND (ESKD OR end-stage kidney disease) yielded no prior reports.

The clinical significance of this case is threefold: it suggests that poor-grade aSAH with ESKD is not an absolute contraindication to treatment; it describes the parameter adjustment strategy of CRRT applied during neurointerventional procedures; and it highlights the significant perioperative bleeding tendency in ESKD patients, emphasizing the importance of airway management.

## Case description

A 43-year-old woman with polycystic kidney disease, polycystic liver disease, and ESKD (CKD stage 5) on maintenance hemodialysis for 5 years, presented with sudden coma (Figures 1A,B). Her last dialysis session was one day prior to admission. She had a history of hypertension and a possible prior SAH two years earlier without vascular imaging.

**Figure 1:**
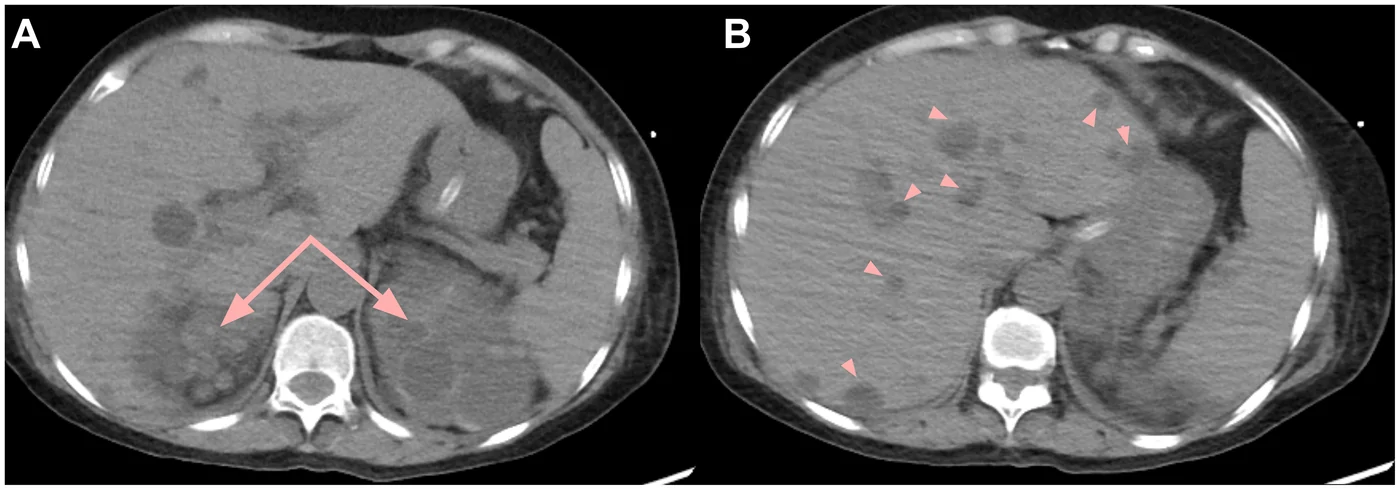
Abdominal CT. **(A)** Pink arrows indicate multiple cysts in both kidneys, consistent with polycystic kidney disease. **(B)** Multiple small pink arrowheads indicate multiple cysts in the liver, consistent with polycystic liver disease.

On admission, she was comatose with GCS 3 (E1V1M1), Hunt-Hess grade V, and anisocoria (left 2.5 mm, right 3.0 mm) with absent light reflexes. CT revealed diffuse SAH (Fisher grade 4), left frontal-temporal intracerebral hematoma, and intraventricular cast formation (Figure 2A). No definite acute hydrocephalus was identified. Laboratory tests showed creatinine 765 μmol/L, eGFR 11.76 mL/min/1.73m^2^, potassium 4.7 mmol/L, platelets 94 × 10⁹/L, and aPTT 41.6 s

**Figure 2:**
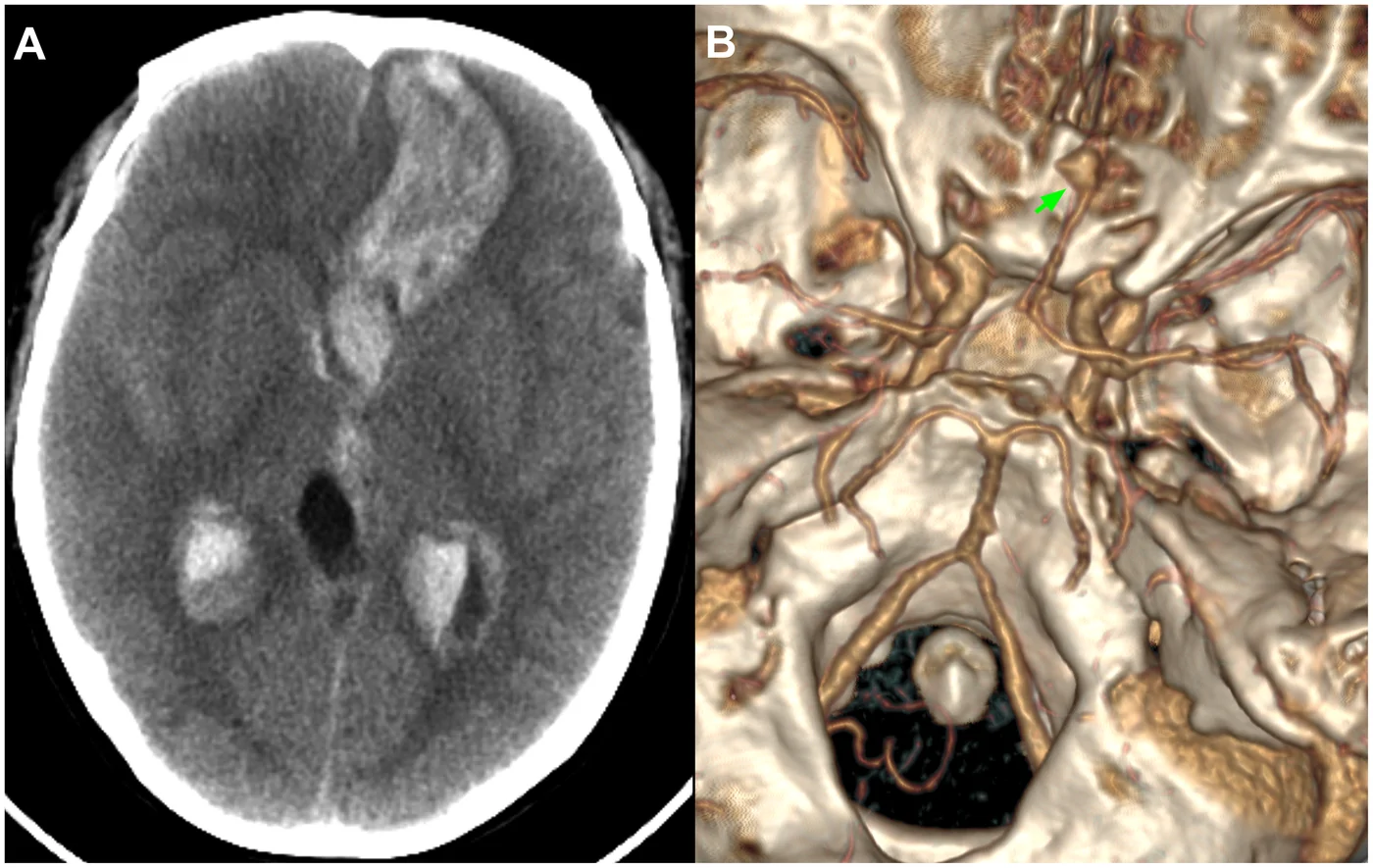
Preoperative imaging. **(A)** Preoperative non-contrast head CT showing diffuse SAH, left frontal-temporal intracerebral hematoma, and intraventricular cast formation. **(B)** Preoperative CTA demonstrating poor contrast opacification of intracranial arteries, consistent with increased intracranial pressure (stasis filling).

Neurocritical care and sedation regimen: The patient was intubated and mechanically ventilated (SIMV mode, tidal volume 6−8 mL/kg, PEEP 5–8 cmH₂O, plateau pressure ≤30 cmH₂O, FiO₂ titrated to maintain SpO₂ ≥ 95%). She received continuous intravenous infusion of propofol (1–2 mg/kg/h), sufentanil (0.2–0.3 μg/kg/h), and midazolam (0.05–0.1 mg/kg/h) for 72 h, after which sedatives were discontinued and analgesia was maintained with sufentanil alone. Mannitol (1 g/kg every 6 h) was administered to maintain serum osmolality between 300 and 320 mOsm/L. Nimodipine (1–2 mg/h) was infused for vasospasm prophylaxis. Bedside hemodialysis was performed on Day 2.

Invasive ICP monitoring and EVD were not performed because the cerebral herniation was primarily caused by the mass effect of the left frontal-temporal hematoma rather than hydrocephalus, and the family refused craniotomy and ventricular drainage. ICP was clinically assessed using hourly pupillary examination and GCS, TCD monitoring of cerebral blood flow, and serial head CT. A total of 1 head CT (on admission) and 1 head and neck CTA (on Day 6 preoperatively) were performed.

Neurological assessment timeline: The first assessment was completed on admission (Day 1). After 72 h of sedation discontinuation, reliable neurological assessment began on Day 5. On Day 6 preoperatively, GCS improved to 7 (E2V1M4) and Hunt-Hess grade improved to IV. The family consented to surgery.

The aneurysm was secured on Day 6. The family initially refused CTA and open surgery, including decompressive craniectomy and ventricular drainage. The patient also required vasoactive support for hypotension on Day 2 and repeated CRRT for severe hyperkalemia. After sedation was discontinued and neurological function improved, the family consented to endovascular treatment. A multidisciplinary discussion preceded transfer to the interventional suite.

Day 6 preoperative laboratory studies: potassium rose sharply to 7.6 mmol/L, platelets 94 × 10⁹/L, aPTT 41.6 s, CRP 141 mg/L. CTA revealed a lobulated aneurysm at the A3 segment of the left anterior cerebral artery (6.0 × 4.1 mm and 3.7 × 2.6 mm) (Figure 2B).

### Simultaneous CRRT and endovascular coiling

Given that potassium 7.6 mmol/L significantly increased the risk of perioperative arrhythmia and cardiac arrest, and that IHD has a significant impact on intracranial pressure and hemodynamics in a patient with cerebral herniation, a multidisciplinary emergency discussion involving neurosurgery, interventional radiology, nephrology, and ICU led to the decision to perform endovascular coiling simultaneously with CRRT.

CRRT was initiated via a right internal jugular temporary dialysis catheter in CVVHDF mode. Initial settings: blood flow rate 150 mL/min, dialysate flow rate 2.0 L/h, replacement fluid flow rate 2.0 L/h, net ultrafiltration rate 1–2 mL/kg/h. The patient's body weight was 74 kg. No anticoagulation was used in the CRRT circuit; saline flushing was performed to maintain patency. During the endovascular procedure, intravenous heparin 0.67 mg/kg was administered separately for the neurointerventional procedure. The net ultrafiltration rate was adjusted every 30 min according to central venous pressure and mean arterial pressure to maintain hemodynamic stability and cerebral perfusion pressure. CRRT continued throughout the 2-hour coiling procedure, with a total replacement/dialysate volume of approximately 8 L. Potassium decreased to 5.2 mmol/L at procedure completion.

Hemodynamic stability: Continuous monitoring of heart rate, blood pressure, and central venous pressure was performed intraoperatively, with dopamine and isoproterenol adjusted as needed to maintain mean arterial pressure >65 mmHg. Intraoperative hemodynamics were stable with no hypotensive events.

Endovascular procedure: Microcatheterization achieved dense coil packing of the aneurysm, with angiography demonstrating complete obliteration (Raymond class I) (Figures 3A,B). Total contrast volume was 85 mL. No stent was placed.

**Figure 3:**
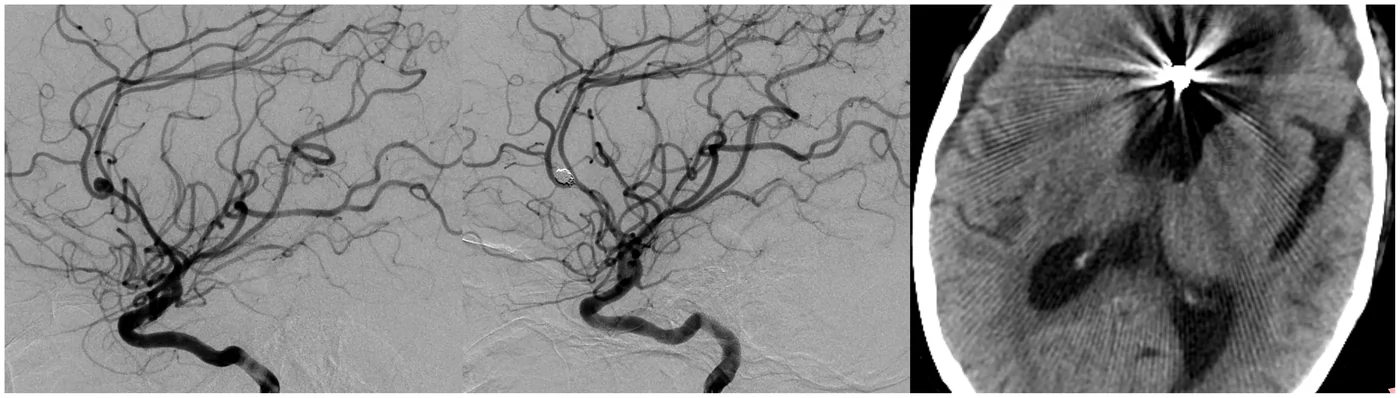
Endovascular treatment and follow-up. **(A)** Preoperative lateral DSA showing a lobulated aneurysm at the A3 segment of the left anterior cerebral artery. **(B)** Post-coiling DSA showing complete obliteration of the aneurysm (Raymond class I). **(C)** Follow-up head CT showing resolution of the frontal lobe and intraventricular hematomas.

### Postoperative management and complications

Postoperatively, the patient gradually regained consciousness (E4M6; verbal component not assessable due to intubation). Repeat head CT showed no new hemorrhage (Figure 3C). Regular hemodialysis was continued.

On postoperative day 8, percutaneous dilatational tracheostomy was attempted due to prolonged ventilator dependence. Preoperatively, the patient was alert and could follow commands, with voluntary movement of both upper extremities, leg movement on the bed, and isometric pupils with light reflexes present. However, refractory bleeding occurred at the puncture site (platelets 89 × 10⁹/L, D-dimer 11.11 μg/mL, FDP 49.3 μg/mL). After platelet transfusion and tranexamic acid, bleeding gradually subsided, but the procedure was aborted. This complication led to airway scarring and crust formation. Despite multiple tube changes, enhanced humidification, and bronchoscopic clearance, recurrent obstruction occurred, and the patient died of acute airway obstruction 3 months postoperatively.

Before tracheostomy, the patient was still within the period of increased vasospasm and delayed cerebral ischemia risk. However, her neurological status was stable, repeat head CT showed no new hemorrhage and no progression of hydrocephalus, and TCD flow velocities had returned to normal. Tracheostomy was therefore performed.

## Discussion

This case involves several key aspects in the management of poor-grade aSAH in ESKD patients, including the timing of aneurysm securing, the prevention of hyperkalemia, the role of CRRT as an intraoperative bridge, the bleeding tendency in ESKD, perioperative airway management, and missed screening opportunities.

Delaying aneurysm securing increases the risk of rebleeding. The POGASH registry reported a pre-treatment rebleeding rate of 17.6% in poor-grade SAH (4). Therefore, early securing is generally preferred. However, in this patient, the initial combination of cerebral herniation, GCS 3, anisocoria, coagulopathy, hypotension, and life-threatening hyperkalemia made immediate intervention extremely risky. After five days of intensive care, the patient's GCS improved from 3 to 7, Hunt-Hess grade improved from V to IV, pupils became isometric, and the family consented to intervention. This allowed a more controlled procedure. The five-day delay may have increased the rebleeding risk, and this is a limitation of this case.

The hyperkalemia in this case may have been largely preventable with scheduled dialysis. Contributing factors included enteral potassium load from daily enteral nutrition emulsion (Ruisu TP) 1,000 mL, stress-induced catabolism after aSAH, only one bedside dialysis session between Day 2 and Day 6 preoperatively, and possible further decline in residual renal function. This should be viewed as a management lesson.

Based on these considerations, we focus on four specific insights from this case. First, CRRT as an intraoperative bridge for poor-grade aSAH with life-threatening hyperkalemia — technical differences from conventional CRRT management. Intraoperative CRRT is well established in prolonged major procedures such as liver transplantation and cardiac surgery. In this patient, CRRT was chosen because of cerebral herniation, intracranial hypertension, and coagulopathy. Short IHD may be a reasonable alternative in patients without these conditions. The most technically significant aspect of this case is the use of CRRT during the endovascular procedure itself, with management details that differ in multiple ways from conventional CRRT. Conventional CRRT in the ICU typically runs continuously for 24 h, uses regional citrate anticoagulation or systemic heparin anticoagulation to maintain filter lifespan, and maintains an ultrafiltration rate of 1–2 mL/kg/h. This case faced three constraints: the need to rapidly clear potassium, the inability to use CRRT circuit anticoagulation due to uremic platelet dysfunction and recent SAH, and the requirement for precise matching with the interventional procedure time window.

These constraints necessitated targeted adjustments to the CRRT prescription. First, no anticoagulation was used in the CRRT circuit; saline flushing was performed to maintain patency. A single intravenous heparin bolus (0.67 mg/kg) was administered separately for the neurointerventional procedure. Second, the net ultrafiltration rate was initiated at 1–2 mL/kg/h and adjusted based on hemodynamic monitoring, while potassium clearance was mainly achieved through dialysate and replacement fluid flows. In this case, parameters were adjusted every 30 min based on MAP and CVP to balance potassium clearance and cerebral perfusion pressure stability. Third, both dialysate and replacement fluid flow rates were increased to 2.0 L/h (total 4.0 L/h), higher than the conventional 1.0–1.5 L/h, achieving a total clearance of approximately 57 mL/kg/h. Fourth, CRRT duration was limited to 2 h, synchronized with the coiling procedure, rather than running continuously for more than 24 h as in conventional practice. Studies have shown that lower net ultrafiltration rates during CRRT help maintain hemodynamic stability, prevent intradialytic hypotension, and preserve cerebral perfusion pressure and brain tissue oxygenation (5). In the case reported by Haryani et al., CVVHDF was run with physiology-guided prescription parameters in a neurocritical care patient, effectively correcting metabolic derangements while maintaining intracranial stability (6). Parameters were adjusted in real time based on bedside monitoring data to ensure the safety of simultaneous procedures.

In this patient, stent-assisted coiling was not used because it would require dual antiplatelet therapy, which was considered high-risk given the elevated intracranial pressure, intracerebral hematoma, uremic platelet dysfunction, and coagulopathy. Simple coil embolization was therefore chosen.

Second, consideration of bleeding tendency — the superimposition of uremic platelet dysfunction and coagulopathy. Uremic platelet dysfunction is an inherent pathological state in ESKD patients, involving altered platelet membrane fluidity, decreased intracellular ADP and serotonin, reduced thromboxane A2 generation, and abnormal GPIIb/IIIa receptor expression. Thekkedath et al. showed that fibrinogen fragment levels in uremic plasma are significantly elevated and inhibit platelet aggregation by competitive binding to the GPIIb/IIIa receptor (7). Baaten et al. noted that bleeding time is prolonged in most CKD patients, platelet adhesion is primarily reduced, and maximum platelet aggregation under collagen stimulation is significantly decreased (1). In this case, admission platelet count was 94 × 10⁹/L and aPTT was 41.6 s, consistent with these changes, though platelet function was not specifically tested. The uremic state can also activate the coagulation system, leading to an imbalance between coagulation and fibrinolysis. ESKD patients often have elevated fibrinogen levels, increased tissue factor expression, and elevated PAI-1 levels that inhibit fibrinolysis. Laboratory data at tracheostomy suggested consumptive coagulopathy: D-dimer rose to 11.11 μg/mL, FDP rose to 49.3 μg/mL, and platelets decreased to 89 × 10⁹/L. Therefore, the bleeding tendency in this case may be the result of uremic platelet dysfunction superimposed with coagulopathy and consumptive coagulopathy, but given that platelet function-specific testing and thromboelastography were not performed, this judgment is only a clinical speculation.

Third, perioperative airway management in ESKD patients requires high priority. The ultimate cause of death in this patient was not the aneurysm but airway management-related complications. Refractory bleeding during tracheostomy led to airway scarring and fatal obstruction. The bleeding risk spectrum constituted by uremic platelet dysfunction and coagulopathy has an impact far beyond the acute neurological event itself. Postoperative monitoring should include not only neurological parameters but also vigilance for coagulation status, airway integrity, and bleeding risk at all invasive sites. Regarding desmopressin, there is currently insufficient evidence for its use in advanced kidney disease (2). In this case, desmopressin was not used prophylactically, and platelet transfusion was the primary approach. Based on this experience, open tracheostomy may be more suitable than percutaneous technique for ESKD patients with coagulopathy, as it allows direct visualization for hemostasis. This hypothesis requires further validation. The timing of tracheostomy in patients still at risk of delayed cerebral ischemia should be individualized.

Fourth, this case highlights a missed screening opportunity. The patient had a possible SAH two years earlier without vascular imaging. A 2025 meta-analysis of 24 studies involving 5,907 ADPKD patients showed that female sex (OR 1.63), family history of IA or SAH (OR 2.17), hypertension (OR 1.41), and CKD stage ≥3 (OR 2.55) are independent risk factors for intracranial aneurysm detection (8). This patient possessed all four risk factors. The 2025 KDIGO guideline supports screening for ADPKD patients with these risk profiles, with non-contrast MRA as the preferred modality (3). For dialysis-dependent ADPKD patients presenting with new headache or suspected SAH, emergent vascular imaging should be pursued.

Regarding the systemic impact of aSAH: Severe aSAH is not merely a localized event in the central nervous system but a systemic disease affecting multiple organ systems. The cardiovascular system in this patient manifested as BNP rising from 289.09 ng/L on admission to 1,032.5 ng/L preoperatively, suggesting neurogenic myocardial stunning. Pulmonary infection was present on admission and worsened postoperatively. Coagulation abnormalities suggested a consumptive coagulopathy component. These multi-system derangements interacted with the patient's underlying ESKD, collectively constituting a high-risk perioperative environment. Ultimately, even though the neurointerventional procedure was technically successful, these non-neurological complications determined the patient's outcome.

### Limitations

First, this is a single case report, and the findings may not be generalizable. Second, the patient did not undergo PKD1/PKD2 genetic testing, invasive ICP monitoring, follow-up vascular imaging, thromboelastography, or platelet function-specific testing. Third, the cause of death was based on clinical judgment, and an autopsy was not performed. Fourth, the five-day delay before aneurysm securing may have increased the rebleeding risk. Fifth, some CRRT prescription parameters, including filter type and filter lifespan, were not available in the original records, and WFNS and modified Rankin Scale were not recorded. Sixth, detailed long-term follow-up records were not available.

## Conclusion

This case suggests that simultaneous CRRT and endovascular coiling was technically feasible in this carefully selected patient with poor-grade aSAH, ESKD, and life-threatening hyperkalemia. Key technical elements included continuous CRRT during the procedure, parameter adjustments differing from conventional CRRT, and multidisciplinary collaboration. However, this is a single-case report and the findings cannot be generalized. In this patient, perioperative bleeding associated with uremic platelet dysfunction and coagulopathy occurred, and airway management was a major challenge. Although the aneurysm was successfully secured and the patient initially recovered neurologically, the subsequent fatal airway complication underscores the complexity of perioperative management in ESKD and shows that endovascular feasibility alone does not guarantee a favorable overall outcome.

### Patient perspective

The patient's daughter, who provided informed consent for this publication, expressed that the family was grateful for the aggressive ICU support that allowed her mother to regain consciousness and communicate with them before her eventual death. She noted that the multidisciplinary team's efforts gave the family additional meaningful time with the patient, and she hoped this case would help other families facing similar situations.

## Data Availability

The original contributions presented in the study are included in the article/Supplementary Material, further inquiries can be directed to the corresponding author.
